# The Southern European Atlantic Diet and all-cause mortality in older adults

**DOI:** 10.1186/s12916-021-01911-y

**Published:** 2021-02-09

**Authors:** Adrián Carballo-Casla, Rosario Ortolá, Esther García-Esquinas, Andreia Oliveira, Mercedes Sotos-Prieto, Carla Lopes, Esther Lopez-Garcia, Fernando Rodríguez-Artalejo

**Affiliations:** 1 Department of Preventive Medicine and Public Health, Universidad Autónoma de Madrid/Idipaz, Calle del Arzobispo Morcillo 4, 28029 Madrid, Spain; 2 CIBER of Epidemiology and Public Health (CIBERESP), Avenida de Monforte de Lemos 3-5, 28029 Madrid, Spain; 3 EPIUnit-Instituto de Saúde Pública da Universidade do Porto, Porto, Portugal; 4 Department of Public Health and Forensic Sciences and Medical Education, Faculty of Medicine, University of Porto, Porto, Portugal; 5 Department of Environmental Health, Harvard T.H. Chan School of Public Health, 665 Huntington Avenue, Boston, MA 02115 USA; 6 IMDEA Food Institute, CEI UAM+CSIC, Carretera de Canto Blanco 8, 28049 Madrid, Spain

**Keywords:** Diet, Mortality, Cohort studies, Proportional hazards models, Elderly

## Abstract

**Background:**

The Southern European Atlantic Diet (SEAD) is the traditional diet of Northern Portugal and North-Western Spain. Higher adherence to the SEAD has been associated with lower levels of some cardiovascular risk factors and reduced risk for myocardial infarction, but whether this translates into lower all-cause mortality is uncertain. We hence examined the association between adherence to the SEAD and all-cause mortality in older adults.

**Methods:**

Data were taken from the Seniors-ENRICA-1 cohort, which included 3165 individuals representative of the non-institutionalized population aged ≥ 60 years in Spain. Food consumption was assessed with a validated diet history, and adherence to the SEAD was measured with an index comprising 9 food components: fresh fish, cod, red meat and pork products, dairy products, legumes and vegetables, vegetable soup, potatoes, whole-grain bread, and wine. Vital status was ascertained with the National Death Index of Spain. Statistical analyses were performed with Cox regression models and adjusted for the main confounders.

**Results:**

During a median follow-up of 10.9 years, 646 deaths occurred. Higher adherence to the SEAD was associated with lower all-cause mortality (fully adjusted hazard ratio [95% confidence interval] per 1-SD increment in the SEAD score 0.86 [0.79, 0.94]; *p*-trend < 0.001). Most food components of the SEAD showed some tendency to lower all-cause mortality, especially moderate wine consumption (hazard ratio [95% confidence interval] 0.71 [0.59, 0.86]). The results were robust in several sensitivity analyses. The protective association between SEAD and all-cause death was of similar magnitude to that found for the Mediterranean Diet Adherence Screener (hazard ratio [95% confidence interval] per 1-SD increment 0.89 [0.80, 0.98]) and the Alternate Healthy Eating Index (0.83 [0.76, 0.92]).

**Conclusions:**

Adherence to the SEAD is associated with a lower risk of all-cause death among older adults in Spain.

**Supplementary Information:**

The online version contains supplementary material available at 10.1186/s12916-021-01911-y.

## Background

The Southern European Atlantic Diet (SEAD) is the traditional dietary pattern of North-Western Spain and Northern Portugal, where staple foods are fish (especially cod), red meat and pork products, dairy, vegetables and potatoes (often eaten as vegetable soup), whole-grain bread, and wine [1–4].

Higher adherence to the SEAD has been associated with a healthier gut microbiota [5] as well as with lower levels of several cardiovascular risk factors, including C-reactive protein, total cholesterol, triglycerides, insulin, insulin resistance, pulse wave velocity, systolic blood pressure, body mass index (BMI), and waist circumference [6–11]. Importantly, higher adherence to the SEAD has also been linked to a lower risk of myocardial infarction [4].

However, some of the SEAD foundations are controversial, as high consumption of red meat and pork products has often been associated with cardiometabolic disease, cancer, and increased mortality [12–14], while consumption of potatoes might increase the risk of type 2 diabetes [15]. Moreover, these characteristics of the SEAD diverge from other healthy dietary patterns that have been consistently linked to a lower risk of chronic diseases and mortality, such as the Mediterranean Diet or the Alternate Healthy Eating Index [16, 17].

Therefore, to better understand the health impact of the SEAD, we used longitudinal data from the Seniors-ENRICA-1 study to examine the association of adherence to the SEAD and its main 9 food components with all-cause mortality in older adults.

## Methods

### Study design and participants

Data came from the Seniors-ENRICA-1 study, a representative cohort of the non-institutionalized persons aged ≥ 60 years in Spain (ClinicalTrials.gov identifier NCT01133093) [18, 19]. The study participants were recruited between March 2008 and September 2010 by stratified cluster sampling. First, the sample was stratified by province and size of the municipality. Second, clusters were selected randomly in 2 stages: municipalities and census sections. Finally, the households within each section were selected by random telephone dialing. Subjects in the households were selected proportionally to the distribution of the population of Spain by sex and age group.

A detailed diet history, a comprehensive set of physical measurements, and a blood test were collected at home visits by trained personnel, whereas data on sociodemographic and lifestyle variables, morbidity, and health services use were gathered through telephone interviews [18]. Study participants were contacted again between February and November 2012 to update information on diet and other study variables and were followed through January 2020 to ascertain vital status. All subjects gave written informed consent, and the Clinical Research Ethics Committee of the “La Paz” University Hospital in Madrid approved the research protocol.

From the 3483 subjects recruited at baseline, we excluded 318 with inadequate data (13 had implausible energy intakes, 239 had incomplete information on diet, and 254 on potential confounders; note that one individual may lack data in more than one variable). Hence, the analytical sample comprised 3165 individuals (Additional file 1: Fig. S1). From these, the 3-year follow-up food consumption was available in 2000 individuals, whereas in 1165, only baseline food consumption was available.

### Study variables

#### Diet

The main exposure variable was 3-year cumulative adherence to the SEAD. Food consumption was obtained in the 2008–2010 and the 2012 visits with a validated electronic diet history [18, 20]. Subjects could report up to 861 foods and recipes habitually consumed in Spain. Portion sizes were estimated with the help of 127 digitized photographs and household measures. Nutrient and energy intake were estimated with Spanish food composition tables [20]. A previous validation study comparing the results of the diet history against seven 24-h recalls over 1 year showed a mean correlation coefficient of 0.53 for all 15 food groups considered, of 0.76 for energy, and of 0.55 for all 41 nutrients studied [20].

To estimate the adherence to the SEAD, we used the food components and scorings proposed by Oliveira et al. [4], which have been used by most of the subsequent studies on this dietary pattern [6–10]. We first calculated the habitual consumption (g/day) of each of the 9 components of this dietary pattern: fresh fish (excluding cod), cod, red meat and pork products, dairy, legumes and vegetables (excluding those consumed in soup), vegetable soup, potatoes regardless of the cooking method, whole-grain bread, and wine. For those study participants who were followed up at 3 years, we averaged the baseline and follow-up food consumption; for those who were not, we used the baseline food consumption. Second, we computed every food component—except wine—as g/1000 kcal/day and calculated their respective sex-specific medians. The subjects who were above the median consumption were scored 1 point, whereas those who were at or below it scored 0 points. As regards wine consumption, men who drank > 0 and ≤ 2 glasses/day and women who drank > 0 and ≤ 1 glasses/day were given 1 point, whereas no points were given for > 2 glasses/day in men, > 1 glass/day in women, or 0 glasses/day. We finally obtained the adherence to the SEAD as the sum of these 9 food group scores; it ranged from 0 to 9, with higher values indicating better adherence.

To place the SEAD into context, we calculated the scores of two other healthy eating patterns: the MEDAS index [21], which reflects the adherence to the Mediterranean diet, and the Alternate Healthy Eating Index (AHEI) [22], whose components were selected based on its association with chronic disease risk. To do this, we first calculated the consumption of the food components of the dietary pattern (14 for the MEDAS and 11 for the AHEI). Second, we scored these according to established cutoff points (scores of 0 or 1 for the MEDAS and 1 to 10 for the AHEI). Third, we summed all components to obtain the final score, which ranges from 0 to 14 for MEDAS and from 0 to 110 for the AHEI; higher values indicate better adherence in both scores (Additional file 1: Table S1).

#### Mortality

On one hand, vital status was ascertained with the National Death Index of Spain, an information system that collects the personal data of every demise recorded on civil registries nationwide [23]. Subjects were matched to the index with combinations of first and last names, birthdates, and national identity card numbers. Hence, the main outcome variable was death from any cause on or before January 31, 2020. Time to death was calculated as the difference between the date of death and the baseline telephone interview.

On the other hand, information on causes of death on or before December 31, 2018, was taken from the National Institute of Statistics of Spain [24]. These data are based on the death certificates of the deceased Spanish residents. Causes of death were classified and grouped according to the International Classification of Diseases (ICD), 10th revision. We considered ICD codes ranging from I00 to I99 to be cardiovascular deaths and those from C00 to D48 to be cancer deaths.

#### Potential confounders

We used data on several potential confounders of the association between the SEAD and mortality, specifically age, sex, educational level (primary or less, secondary, or university), energy intake (kcal/day), tobacco smoking (never, former, or current), recreational physical activity, time spent watching TV (h/day) (as a proxy of sedentary behavior) [25], BMI, and morbidity. Physical activity was assessed with the validated questionnaire developed in the EPIC study in Spain [26] and expressed as metabolic equivalents of task hours/week [27]. TV hours/day were assessed with the Nurses’ Health Study questionnaire validated in Spain [28]. BMI was calculated as weight (kg) divided by height (m) squared, both measured under standardized conditions [29]. As regards morbidity, we considered that a subject had diabetes if he/she either had blood glucose levels ≥ 126 mg/dl, was being treated with antidiabetic drugs, or reported that their doctor had given them a diabetes diagnosis. The medical diagnoses of cardiovascular disease (heart attack, stroke, or heart failure), chronic obstructive respiratory disease, musculoskeletal disease (osteoarthritis, arthritis, or hip fracture), cancer, and depression requiring medical treatment also were self-reported.

### Statistical analyses

Differences in baseline characteristics and nutrient intakes of study participants across categories of the SEAD score were evaluated with Pearson’s chi-squared tests for discrete variables and analysis of variance for continuous variables.

The association between SEAD adherence and all-cause mortality was summarized with hazard ratios (HR), and their 95% confidence interval (CI), estimated with Cox proportional hazards regression. To control for potential confounding, three incrementally adjusted models were used: (1) adjusted for sociodemographic characteristics (age, sex, and educational level) and energy intake, (2) additionally adjusted for lifestyle variables and morbidity (tobacco smoking, physical activity, time watching TV, BMI, diabetes, cardiovascular disease, respiratory disease, musculoskeletal disease, cancer, and depression), and (3) further adjusted for the consumption of common foods not included in the SEAD (white meat, fruits, and refined grains). In the analyses, we used baseline values for categorical variables and averaged the baseline and 3-year follow-up values for continuous variables.

The adherence to the SEAD was modeled in the analyses as (1) a continuous variable (per 1-SD increment), (2) quartiles (using the lowest one as the reference), and (3) a restricted cubic spline (knots located at 2.5, 3.5, and 4.5 points). The adherence to the MEDAS and AHEI scores was modeled alike. When we examined the individual food components comprising the SEAD and the MEDAS, they were entered into the models as dichotomous variables (above or below specific food consumption thresholds). Conversely, components of the AHEI were modeled as continuous variables. Further details can be found in Additional file 1: Table S1.

We conducted several sensitivity analyses: First, we calculated the adherence to the 9 SEAD food components in two other different ways: (1) scoring 1, 2, 3, or 4 points if subjects were respectively in the 1st, 2nd, 3rd, or 4th sex-specific quartile of the consumption of the food component (in g/1000 kcal/day) and (2) scoring 1 point if subjects consumed < 1 serving/week of the food component (in g/week), 2 points for 1 to 7 servings/week, and 3 points for ≥ 1 serving/day. Second, we calculated the SEAD adherence with reverse scoring for the consumption of red meat and pork products and for potatoes, as higher consumption of these foods may have deleterious health effects [12–15]. For each of these two food components, subjects who were above the sex-specific median consumption were hereby scored 0 points, whereas those who were at or below it received 1 point. Third, to better understand the contribution of alcohol intake to the association between the SEAD and mortality, we further calculated the SEAD adherence without scoring wine consumption. Fourth, to reduce the potential residual confounding regarding morbidity, we adjusted the analyses for blood pressure- and lipid-lowering drugs, which are two of the most habitually used chronic medications. Fifth, to minimize the potential for reverse causation—health status influencing food consumption, rather than the opposite—we alternatively excluded from the analyses the subjects who died within the first 2 years of follow-up and those with prevalent morbidity (diabetes, cardiovascular disease, respiratory disease, musculoskeletal disease, cancer, or depression). And sixth, to test the consistency of our results with those for the main causes of death, we replicated the analyses for cardiovascular disease and cancer mortality.

Lastly, we conducted two additional analyses to address a potential methodological limitation: to minimize measurement error in SEAD adherence, we averaged the baseline and follow-up food consumption values for those participants who were followed at 3 years, but we used the baseline food consumption for those who were not, and who likely had worse health and higher mortality (for some of them had already died at this follow-up wave) than the subjects who remained in the cohort. To investigate how loss to follow-up may have affected our findings, we first compared the baseline characteristics between participants who were and were not followed at 3 years. Secondly, to test if the association of SEAD with mortality differed between the subjects who were and were not followed up at 3 years, we calculated the hazard ratio of the multiplicative interaction as follows: HR^int^ = HR(SEAD^+^ Follow-up^+^)/[HR(SEAD^+^ Follow-up^−^) × HR(SEAD^−^ Follow-up^+^)].

To assure that point estimates and their confidence intervals were representative of the Spanish population, descriptive data and regression analyses accounted for the complex sampling design, using the *svy* command in Stata® (StataCorp LLC), version 14.0.

## Results

### Descriptive and outcome data

Characteristics of the study participants and information on potential confounders are shown in Table 1. Compared to individuals in the lowest quartile of the SEAD score, those in the higher quartiles had a higher educational level and BMI and a healthier overall lifestyle (were less likely to smoke, did more physical activity, and had higher consumption of white meat and fruit and lower consumption of refined grains). Regarding morbidity, their prevalence of diabetes and cancer was higher, but they suffered less from depression. As for nutrient intake (Additional file 1: Table S2), higher SEAD adherence was correlated with higher total protein and animal protein intake, lower total fat and saturated fat intake, increased omega-3 fatty acid intake, reduced carbohydrate intake, higher fiber intake, and higher vitamin (carotenoids, B_1_, B_6_, C, and E) and mineral (potassium, calcium, iron, and selenium) intake.

**Table 1:** Characteristics of the study participants by adherence to the Southern European Atlantic Diet (SEAD)

	SEAD score^**a**^ (quartiles)				
	1	2	3	4	Missing
**Deaths**^**b**^	181	159	145	162	77
***n***	688	761	787	929	318
Sex—men^c^ (%)	40.1	46.8	46.9	47.5	42.1
Age^c^	70.3 (6.9)	69.8 (6.9)	69.8 (6.6)	69.5 (6.2)	71.0 (7.6)
Educational level^c^ (%)					
Primary or less	66.0	58.2	61.1	53.1*	61.1
Secondary	22.4	22.2	22.0	25.3	20.8
University	11.6	19.6	16.9	21.6	18.1
Tobacco smoking^c^ (%)					
Never	60.2	54.9	59.5	59.4*	61.6
Former	26.2	31.1	31.2	30.5	23.8
Current	13.5	14.0	9.3	10.0	14.5
Diabetes^c^ (%)	18.0	12.8	19.8	20.3*	21.6
Cardiovascular disease^c^ (%)	7.61	4.33	7.22	4.96	7.05
Respiratory disease^c^ (%)	8.82	7.85	9.68	6.77	6.32
Musculoskeletal disease^c^ (%)	53.0	47.4	51.8	49.2	49.3
Cancer^c^ (%)	2.85	1.61	1.17	3.96*	2.48
Depression^c^ (%)	12.1	7.4	9.9	5.8*	11.7
Leisure-time physical activity^d^ (MET-hours/week)	19.8 (13.4)	20.7 (13.2)	21.5 (13.5)	22.9 (14.1)*	19.7 (14.2)
Time watching TV^d^ (h/day)	2.89 (1.72)	2.72 (1.47)	2.77 (1.49)	2.67 (1.40)	2.88 (1.82)
Body mass index^d^ (kg/m^2^)	28.2 (4.3)	28.4 (4.3)	29.0 (4.6)	28.7 (4.3)*	28.7 (5.3)
Energy intake^d^ (kcal/day)	2014 (531)	1965 (492)	1952 (464)	1934 (453)	1853 (594)
White meat consumption^d^ (g/day)	33.3 (30.4)	33.7 (29.3)	37.8 (28.9)	38.5 (27.0)*	31.6 (30.0)
Fruit consumption^d^ (g/day)	296 (181)	324 (167)	338 (176)	341 (167)*	310 (182)
Refined grains consumption^d^ (g/day)	223 (92)	206 (93)	202 (92)	177 (85)*	204 (95)

Values are means (standard deviations) unless otherwise indicated. *p value < 0.05 for differences in means (ANOVA) or proportions (Pearson’s chi-squared) across categories of the SEAD score

^a^Sex-specific medians were used as the threshold for all food components, except wine. Quartile values of the SEAD score: quartile 1, ≤ 2; quartile 2, 3; quartile 3, 4; quartile 4, ≥ 5

^b^The number of deaths/1000 person-years [95% confidence interval] for increasing quartiles of the SEAD score was 26.8 [22.6, 32.1], 20.5 [17.2, 24.7], 17.9 [15.0, 21.6], and 16.9 [14.2, 20.2], respectively

^c^At baseline

^d^Three-year cumulative values (baseline and 3-year follow-up if available)

During a median follow-up of 10.9 years (32,158 person-years of follow-up), 646 subjects died. The number of deaths/1000 person-years [95% CI] for increasing quartiles of SEAD score was 26.8 [22.6, 32.1], 20.5 [17.2, 24.7], 17.9 [15.0, 21.6], and 16.9 [14.2, 20.2], respectively.

### Main results

**Fig. 1:**
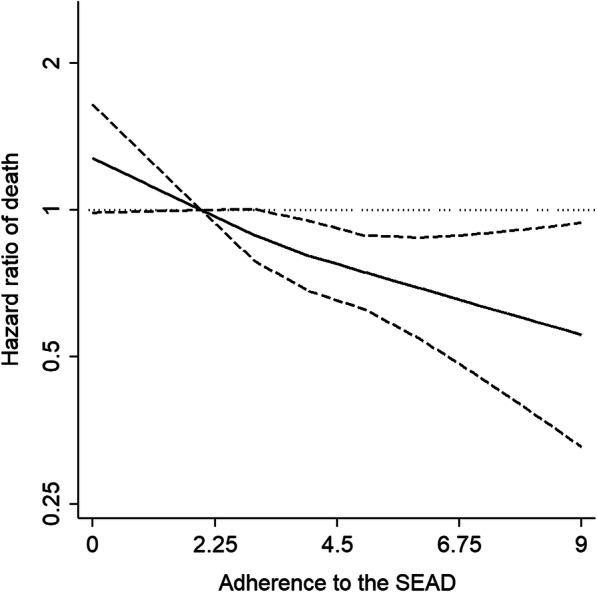
Association between adherence to the Southern European Atlantic Diet (SEAD) and risk of all-cause death. Plotted values are hazard ratios (95% confidence intervals) from a Cox proportional hazards model as model 2 in Table 2, adjusted for sex, age (years), educational level (primary or less, secondary, or university), smoking status (never, former, or current), diabetes, cardiovascular disease, respiratory disease, musculoskeletal disease, cancer, and depression at baseline, and 3-year cumulative leisure-time physical activity (MET-hours/week), sedentary behavior (TV hours/day), body mass index (kg/m^2^), and energy intake (kcal/day). The restricted cubic spline knots are located at 2.5, 3.5, and 4.5 points of adherence to the SEAD score

The association between adherence to the SEAD and all-cause mortality is shown in Table 2. Higher adherence to the SEAD was consistently associated with a lower risk of death (model 2 HR [95% CI] per 1-SD increment in the SEAD score = 0.86 [0.79, 0.94]), with a clear dose-response (Fig. 1; *p*-trend < 0.001). Most individual food components of the SEAD showed some tendency to lower all-cause mortality, but associations were generally weak except for moderate wine consumption (model 2 HR [95% CI] = 0.71 [0.59, 0.86]) (Table 3).

**Table 2:** Association between adherence to the Southern European Atlantic Diet (SEAD) score and all-cause death (*n* = 3165)

	Deaths	Person-years	Hazard ratio [95% CI]		
			Model 1	Model 2	Model 3
**SEAD score based on sex-specific medians of food consumption**					
Per 1-SD increment	646	32,158	0.85 [0.78, 0.93]***	0.86 [0.79, 0.94]**	0.86 [0.78, 0.94]***
Quartiles^a^					
1 (lower adherence)	181	6728	1	1	1
2	159	7733	0.79 [0.60, 1.04]	0.83 [0.64, 1.08]	0.83 [0.64, 1.09]
3	145	8084	0.63 [0.48, 0.83]***	0.66 [0.50, 0.87]**	0.66 [0.50, 0.87]**
4 (higher adherence)	162	9613	0.66 [0.52, 0.85]**	0.68 [0.53, 0.88]**	0.67 [0.52, 0.87]**
**Sensitivity analyses**					
**SEAD score based on sex-specific quartiles of food consumption**					
Per 1-SD increment	646	32,158	0.88 [0.80, 0.96]**	0.87 [0.80, 0.96]**	0.86 [0.79, 0.95]**
Quartiles^b^					
1 (lower adherence)	197	7693	1	1	1
2	192	9800	0.81 [0.64, 1.03]	0.81 [0.64, 1.03]	0.81 [0.64, 1.03]
3	110	6389	0.65 [0.50, 0.86]**	0.68 [0.51, 0.89]**	0.67 [0.51, 0.88]**
4 (higher adherence)	147	8276	0.70 [0.55, 0.89]**	0.70 [0.55, 0.90]**	0.68 [0.53, 0.88]**
**SEAD score based on frequency of food consumption**					
Per 1-SD increment	646	32,158	0.80 [0.73, 0.89]***	0.79 [0.71, 0.87]***	0.78 [0.70, 0.86]***
Quartiles^c^					
1 (lower adherence)	193	6863	1	1	1
2	156	8060	0.76 [0.59, 0.97]*	0.74 [0.58, 0.96]*	0.75 [0.58, 0.96]*
3	166	8043	0.77 [0.60, 0.98]*	0.76 [0.59, 0.97]*	0.76 [0.60, 0.97]*
4 (higher adherence)	132	9192	0.61 [0.47, 0.80]***	0.60 [0.46, 0.78]***	0.58 [0.44, 0.76]***

*CI* confidence interval

*p < 0.05; **p < 0.01; ***p < 0.001

^a^Quartile values of the SEAD score: quartile 1, ≤ 2; quartile 2, 3; quartile 3, 4; quartile 4, ≥ 5. Range [0, 9]

^b^Quartile values of the SEAD score: quartile 1, ≤ 15; quartile 2, 16 to 18; quartile 3, 19 to 20; quartile 4, ≥ 21. Range [9, 32]

^c^Quartile values of the SEAD score: quartile 1, ≤ 14; quartile 2, 15; quartile 3, 16; quartile 4, ≥ 17. Range [10, 22]

Model 1: Cox proportional hazards model adjusted for sex, age (years), educational level (primary or less, secondary, or university), and energy intake (kcal/day)

Model 2: as model 1 and further adjusted for smoking status (never, former, or current), diabetes, cardiovascular disease, respiratory disease, musculoskeletal disease, cancer, and depression at baseline, and 3-year cumulative leisure-time physical activity (MET-hours/week), sedentary behavior (TV hours/day), and body mass index (kg/m^2^)

Model 3: as model 2 and further adjusted for 3-year cumulative white meat, fruit, and refined grains consumption

**Table 3:** Association between adherence to the food components of the Southern European Atlantic Diet (SEAD) and risk of death from any cause (*n* = 3165)

	Median consumption (g/1000 kcal)				
Food component	Women	Men	Deaths	Person-years	Hazard ratio [95% CI]^**a**^
**Fresh fish (excluding cod)**	26.5	26.3			
≤ Median			352	16,004	Ref.
> Median			294	16,154	0.91 [0.75, 1.09]
**Cod**	0	0			
≤ Median			508	24,940	Ref.
> Median			138	7218	0.92 [0.76, 1.13]
**Red meat and pork products**	24.0	27.3			
≤ Median			348	15,949	Ref.
> Median			298	16,209	0.98 [0.80, 1.19]
**Dairy products**	178	132			
≤ Median			282	16,279	Ref.
> Median			365	15,878	1.02 [0.85, 1.23]
**Legumes and vegetables**	124	119			
≤ Median			367	15,836	Ref.
> Median			279	16,322	0.86 [0.72, 1.04]
**Vegetable soup**	0	0			
≤ Median			467	23,620	Ref.
> Median			180	8538	0.97 [0.79, 1.19]
**Potatoes**	19.9	22.6			
≤ Median			325	16,095	Ref.
> Median			321	16,063	0.93 [0.77, 1.11]
**Whole-grain bread**	0	0			
≤ Median			523	23,870	Ref.
> Median			123	8288	0.80 [0.63, 1.02]
**Wine (glasses/day)**	0	0.42			
0 or > 1 glass/day (women), 0 or > 2 glasses/day (men)			385	17,018	Ref.
≥ 0 to 1 glass/day (women), ≥ 0 to 2 glasses/day (men)			261	15,139	0.71 [0.59, 0.86]***

*CI* confidence interval

*p < 0.05; **p < 0.01; ***p < 0.001

^a^Cox proportional hazards model as model 2 in Table 2, adjusted for sex, age (years), educational level (primary or less, secondary, or university), smoking status (never, former, or current), diabetes, cardiovascular disease, respiratory disease, musculoskeletal disease, cancer, and depression at baseline, and 3-year cumulative leisure-time physical activity (MET-hours/week), sedentary behavior (TV hours/day), body mass index (kg/m^2^), and energy intake (kcal/day)

The protective association between the SEAD and all-cause death was of similar magnitude to that found for the MEDAS (model 2 HR [95% CI] per 1-SD increment = 0.89 [0.80, 0.98]; *p*-trend = 0.005) and the AHEI (0.83 [0.76, 0.92]; *p*-trend < 0.001). (Additional file 1: Table S3 and Fig. S2). The food components of these two dietary patterns that showed the strongest associations with lower mortality were high consumption of olive oil, nuts, and vegetable sauce and low consumption of sugar-sweetened beverages for the MEDAS, and high intake of n-3 fatty acids, moderate intake of alcohol, and low consumption of sugar-sweetened beverages for the AHEI (Additional file 1: Table S4).

### Other analyses

Results from the sensitivity analyses were as follows: (1) when calculating the SEAD score based on sex-specific quartiles of food consumption instead of sex-specific medians, the association with mortality remained similar, and it even strengthened when the SEAD score was calculated based on the frequency of food consumption (Table 2). The associations were also robust when (2) calculating the SEAD with reverse scoring for red meat and pork products and for potatoes (model 2 HR [95% CI] per 1-SD increment = 0.89 [0.81, 0.98]), when (3) calculating the SEAD without considering wine consumption (0.91 [0.83, 0.99]), when (4) adjusting the analyses for the consumption of blood pressure- and lipid-lowering drugs (0.87 [0.80, 0.95]), and when (5) excluding from the analyses the 50 subjects who died within the first 2 years of follow-up (0.89 [0.81, 0.97]) or the 2051 participants with chronic diseases at baseline (0.75 [0.63, 0.91]). Finally, adherence to the SEAD showed some tendency to reduced cardiovascular and cancer mortality, which did not reach statistical significance (0.84 [0.69, 1.02] and 0.90 [0.75, 1.08], respectively).

Compared to the participants who were followed up at 3 years, those who were lost were more likely to be women (53.7% vs 59.6%), slightly older (69.4 vs 70.2 years), less educated (19.7% vs 12.0% had university studies), and more often suffered from depression (7.9% vs 11.2%). However, the association of adherence to the SEAD and risk of death was similar among the subjects who were and were not followed up (HR of the multiplicative interaction [95% CI] = 1.17 [0.79, 1.74]).

## Discussion

### Key findings

In this cohort, representative of the older adult population of Spain, higher adherence to the SEAD was consistently associated with reduced all-cause mortality. The associations between the food components of the SEAD and lower all-cause mortality were weak, except for moderate wine consumption. The protective association between SEAD adherence and all-cause death was of similar magnitude to that found for the MEDAS or the AHEI.

### Interpretation

Our results are in line with those of previous studies regarding the SEAD. In a cross-sectional study in younger adults from North-Western Spain, higher SEAD adherence was associated with a higher quantity of *Bifidobacterium* in feces, a probiotic genus which is thought to be important for physiological functions, such as the development of the host immune response [5]. Also, in another cross-sectional study representative of the Spanish population ≥ 18 years, higher adherence to the SEAD was associated with lower levels of C-reactive protein, triglycerides, insulin, insulin resistance, and systolic blood pressure. The individual components of the SEAD that mostly mediated these beneficial associations were fish and legumes/vegetables for C-reactive protein, fish for triglycerides, and cod and legumes/vegetables for blood pressure [6]. These findings were replicated and expanded in another cross-sectional study in Portuguese adolescents, as increased SEAD adherence was also associated with lower total cholesterol, as well as with a smaller waist circumference [7–10]. In a further cross-sectional study of morbidity-free Spanish subjects < 70 years, higher adherence to a modified 14-component SEAD score that included physical activity was associated with lower levels of total cholesterol, triglycerides, pulse wave velocity, BMI, and waist circumference [11]. Finally, in a case-control study in Portuguese adults ≥ 18 years, higher SEAD adherence was linked to lower odds of non-fatal acute myocardial infarction. The food groups that contributed the most to this association were cod, dairy products, legumes and vegetables, whole-grain bread, and wine. Conversely, consumption of red meat and pork products as well as of potatoes was associated with higher odds of myocardial infarction [4].

Although no studies outside Spain and Portugal have examined the association of adherence to the SEAD with mortality, the effects of individual SEAD food components on all-cause mortality have indeed been studied in other countries. Two recent dose-response meta-analyses demonstrated a beneficial association for fish, legumes, vegetables, and whole-grains consumption; a detrimental association for red and processed meat consumption; and no association for dairy or potatoes [14, 15]. Also, there seem to be no clear mortality benefits from moderate alcohol intake, although wine might have a distinct effect because it rarely entails binge drinking [30, 31]. Moreover, increased adherence to the SEAD was correlated in our study with a nutrient pattern that has been linked to lower risk of death: (1) decreased saturated fat and increased omega-3 fatty acid intakes [32, 33], (2) increased protein intake (although its effects on mortality could be opposite in older and younger subjects) [34–36], (3) increased fiber intake [37], and (4) increased vitamin and mineral intake (but not of sodium) [38–41].

Although we have found a significant association between the SEAD and lower mortality, we have been unable to demonstrate as much for the individual food groups contributing to it -except for wine consumption-. Moreover, a SEAD score calculated with reverse scoring for red and processed meat and for potatoes was not more strongly associated with lower mortality risk than the original SEAD score. Any explanation for these findings must be conjectural. On one hand, dietary patterns can account for the small cumulative effects of individual foods on chronic disease and for complex interactions between food components [42]. On the other hand, cooking techniques might account for part of the effects on health that are sometimes attributed to foods themselves. Namely, while fried potato consumption has been associated with increased risk for all-cause mortality, type 2 diabetes, and hypertension, non-fried potatoes—which were consumed 5 times as much by our study participants—seem to have a negligible influence on these outcomes [15]. It is also possible that some foods have a distinct health effect on younger and older subjects. For example, the consumption of red and processed meat is likely to be lower in the elderly than in younger adults, which could minimize its deleterious associations with cardiovascular and cancer risk [12, 13]. Moreover, the high-quality protein content of these foods may help delay sarcopenia, a common cause of physical disability [34, 35, 43]. Lastly, the SEAD scoring for wine consumption does not account for potential biases regarding alcohol intake, such as the abstainer bias, the healthy drinker/survivor bias, or reverse causation [31]. This might explain the observed favorable association of moderate wine consumption and mortality, as 50% of our study subjects who were given 0 points for this component were never drinkers and 21% were former drinkers.

### Generalizability

How applicable are our estimates there where the SEAD is the traditional dietary pattern, specifically in Northern Portugal and North-Western Spain? As regards Northern Portugal, a case-control study showed that 50.1% of the controls, which were representative of the population of Porto, had a high SEAD adherence (score based on the frequency of food consumption ≥ 18) [4], opposite to 10.0% of subjects from our study. Within our participants, there were also differences when comparing subjects from North-Western Spain with those from other Spanish regions (16.2% vs 9.5% had a SEAD score based on the frequency of food consumption ≥ 18). Nevertheless, the dose-response relationship for the association between the SEAD and mortality was strong at any level of adherence to the SEAD (Fig. 1), and it was not significantly different between the subjects who lived in North-Western Spain and those who did not (HR of the multiplicative interaction [95% CI] = 0.72 [0.33, 1.56]).

We should also consider that our study comprised people ≥ 60 years, so the results may not necessarily apply to younger populations. On one hand, if the association between the SEAD and mortality also operated before the study onset, the selection of older subjects could bias the estimates towards the null, as participants who had survived so far despite their lower adherence to the SEAD were probably less likely to die from any cause, regardless of their diet. On the other hand, the study association could be stronger in older than in younger subjects due to increased cumulative exposure to the diet in the years or decades before enrollment. It is therefore reassuring that our results are in line with those of other studies on the SEAD carried out in younger, Portuguese, and North-Western Spanish populations [4–11].

### Limitations

As in other studies in nutritional epidemiology, some limitations in diet assessment should be acknowledged. The correlation for food consumption between our diet history and seven 24-h recalls through 1 year was moderate (*r* = 0.53), though similar to that of other methods used to measure habitual diet in and out of Spain [20]. To better measure diet, we averaged the baseline and 3-year follow-up food consumption for those subjects with two diet records. In any case, the inability to measure the true value of a dietary exposure would usually bias the study results towards unity: the greater the imprecision, the greater the bias [44], so we would likely be underestimating the true association between the SEAD and mortality.

A further limitation is that many covariates—including the medical diagnoses of cardiovascular disease, chronic obstructive respiratory disease, musculoskeletal disease, cancer, and depression—were self-reported. Despite using validated questions and scales from previous health surveys [18, 45], they too have been measured with some error. Moreover, the diagnosis of diabetes did not use data on glycated hemoglobin, which might have underestimated the number of cases. The real range of uncertainty in estimates could then be larger than that reflected in confidence intervals, for when exposures and several confounders are measured with various degrees of precision, the adjusted hazard ratios could be biased in any direction [46]. We cannot rule out residual confounding either, despite adjusting the models for many sociodemographic, lifestyle, and diet-related variables as well as morbidity. It is encouraging though to see how close the results from minimally adjusted and fully adjusted models are from one another.

## Conclusions

In the older adult population of Spain, higher adherence to the SEAD is associated with lower long-term mortality. The reduced risk of all-cause death associated with the SEAD is consistent in both main and sensitivity analyses, and similar to that of other healthy dietary patterns, such as the Mediterranean diet or the AHEI. Nevertheless, diet can change over time and its effects on mortality could be cumulative and have long induction periods [47], so more evidence from studies with repeated measurements of diet and longer-term follow-ups is still needed. Further research should also assess the effect of the SEAD in populations other than those of Spain and Portugal.

## Supplementary Information

**Additional file 1: Table S1.** Items and scoring of the Mediterranean Diet Adherence Screener (MEDAS) and the Alternate Healthy Eating Index (AHEI). **Table S2.** Nutrient intakes by adherence to the Southern European Atlantic Diet (SEAD). **Table S3.** Association between adherence to the Mediterranean Diet Adherence Screener (MEDAS) and the Alternate Healthy Eating Index (AHEI), and risk of all-cause death (*n*= 3165). **Table S4.** Association between adherence to the items of the Mediterranean Diet Adherence Screener (MEDAS) and the Alternate Healthy Eating Index (AHEI), and risk of all-cause death (n= 3165). **Figure S1.** Participants’ flow chart. **Figure S2.** Association between adherence to the Mediterranean Diet Adherence Screener (MEDAS) and the Alternate Healthy Eating Index (AHEI), and risk of all-cause death.

## Data Availability

The datasets used and/or analyzed during the current study are available from the corresponding authors on reasonable request.

## Abbreviations

AHEI: Alternate Healthy Eating Index
BMI: Body mass index
CI: Confidence interval
HR: Hazard ratio
MEDAS: Mediterranean Diet Adherence Screener
SEAD: Southern European Atlantic Diet
