# Supplementary material for: The Southern European Atlantic Diet and all-cause mortality in older adults
Source: BMC Med. 2021 Feb 9;19:36. doi: 10.1186/s12916-021-01911-y (PMC7871632; doi:10.1186/s12916-021-01911-y)
Supplement: Supplementary file 1 — Additional file 1: Table S1. Items and scoring of the Mediterranean Diet Adherence Screener (MEDAS) and the Alternate Healthy Eating Index (AHEI). Table S2. Nutrient intakes by adherence to the Southern European Atlantic Diet (SEAD). Table S3. Association between adherence to the Mediterranean Diet Adherence Screener (MEDAS) and the Alternate Healthy Eating Index (AHEI), and risk of all-cause death (n= 3165). Table S4. Association between adherence to the items of the Mediterranean Diet Adherence Screener (MEDAS) and the Alternate Healthy Eating Index (AHEI), and risk of all-cause death (n= 3165). Figure S1. Participants’ flow chart. Figure S2. Association between adherence to the Mediterranean Diet Adherence Screener (MEDAS) and the Alternate Healthy Eating Index (AHEI), and risk of all-cause death. [file 12916_2021_1911_MOESM1_ESM.docx]

**Table S1.** Items and scoring of the Mediterranean Diet Adherence Screener (MEDAS) and the Alternate Healthy Eating Index (AHEI).

| **Mediterranean Diet Adherence Screener (MEDAS)** | **Minimum score (0)** | **Maximum score (1)** |
| --- | --- | --- |
| 1. Using olive oil as the main source of fat for cooking | No | Yes |
| 1. Olive oil (servings/day) | <4 | ≥4 |
| 1. Vegetables (servings/day for low/int./high energy intake) | <2/2.33/2.67 | ≥2/2.33/2.67 |
| 1. Fruit, including fresh-squeezed juice (servings/day for low/int./high energy intake) | <3/3.6/4.2 | ≥3/3.6/4.2 |
| 1. Red and processed meats (servings/day for low/int./high energy intake)c | ≥0.8/1/1.2 | <0.8/1/1.2 |
| 1. Butter, margarine, or cream (servings/day for low/int./high energy intake) | ≥0.83/1/1.25 | <0.83/1/1.25 |
| 1. Carbonated or sugar-sweetened beverages (servings/day) | ≥1 | <1 |
| 1. Wine (servings/week for low/int./high energy intake) | <4.67/5.83/7 | ≥4.67/5.83/7 |
| 1. Legumes (servings/week for low/int./high energy intake) | <3/3.5/4 | ≥3/3.5/4 |
| 1. Fish or seafood (servings/week for low/int./high energy intake) | <3/3.76/4.5 | ≥3/3.76/4.5 |
| 1. Commercial pastries (servings/week for low/int./high energy intake) | ≥2/3/4 | <2/3/4 |
| 1. Nuts (servings/week for low/int./high energy intake) | <2/2.5/3 | ≥2/2.5/3 |
| 1. Preferring white meat over red meat | No | Yes |
| 1. Dishes with a traditional sauce of tomatoes, garlic, onion, or leeks sautéed in olive oil (servings/week) | <2 | ≥2 |
| **TOTAL** | 0 | 14 |

**Table S1 (cont.)**

| **Alternate Healthy Eating Index (AHEI)** | **Minimum score (0)** | **Maximum score (10)** |
| --- | --- | --- |
| 1. Vegetables, excluding potatoes (servings/day) | 0 | ≥5 |
| 1. Fruit (servings/day) | 0 | ≥4 |
| 1. Whole grains (g/day) | 0 | ≥75 (women), ≥90 (men) |
| 1. Nuts and legumes (servings/day) | 0 | ≥1 |
| 1. Long-chain n-3 fats (EPA+DHA) (mg/day) | 0 | ≥250 |
| 1. Polyunsaturated fatty acids (% of energy intake) | ≤2 | ≥10 |
| 1. Sugar-sweetened beverages and fruit juice (servings/day) | ≥1 | 0 |
| 1. Red and processed meats (servings/day) | ≥1.5 | 0 |
| 1. Trans fat (% of energy intake) | ≥0 | ≤0.5 |
| 1. Sodium (mg/day) | Highest decile | Lowest decile |
| 1. Alcohol (g/day) | ≥25 (women), ≥35 (men) | 5-15 (women), 5-20 (men) |
| **TOTAL** | 0 | 110 |
| For the scoring of MEDAS: 1 serving of olive oil = 9 g; 1 serving of vegetables = 150 g; 1 serving of fruit = 125 g; 1 serving of red/processed meats = 125 g; 1 serving of butter, margarine or cream = 12 g; 1 serving of carbonated or sugar-sweetened beverages = 200 mL; 1 serving of wine = 100 mL; 1 serving of legumes = 150 g; 1 serving of fish or seafood = 100 g; 1 serving of commercial pastries = 50 g; 1 serving of nuts = 30 g. | | |
| For the scoring of AHEI: Intakes between the minimum (0) and maximum (10) levels are scored proportionately. 1 serving of vegetables = 0.5 cups of vegetables or 1 cup of green leafy vegetables; 1 serving of fruit = 160 g; 1 serving of sugar-sweetened beverages and fruit juice = 8 oz; 1 serving of red/processed meats = 4 oz of unprocessed meat or 1.5 oz of processed meat (1 cup = 236.59 g; 1 oz = 28.35 g). Values in the lowest decile of sodium were ≤1887 mg/day in men and ≤1455 mg/day in women, and in highest decile were ≥4260 mg/day in men and ≥3467 mg/day in women. Non-drinkers received a score of 2.5. | | |

**Table S2.** Nutrient intakes by adherence to the Southern European Atlantic Diet (SEAD).

|  | **SEAD score ^a^ (quartiles)** | | | | |
| --- | --- | --- | --- | --- | --- |
|  | **1** | **2** | **3** | **4** | **Missing** |
| **Deaths ^b^** | 181 | 159 | 145 | 162 | 77 |
| **n** | 688 | 761 | 787 | 929 | 318 |
| Total Protein (g/day) | 85.0 (23.2) | 87.4 (22.8) | 90.7 (21.5) | 93.3 (21.0)^*^ | 86.6 (36.9) |
| Animal protein (g/day) | 55.0 (19.0) | 57.6 (19.2) | 60.7 (17.4) | 64.0 (17.1)^*^ | 58.0 (26.1) |
| Plant protein (g/day) | 29.9 (9.54) | 29.9 (8.96) | 30.0 (8.68) | 29.3 (8.17) | 28.5 (13.2) |
| Total fat (g/day) | 82.7 (28.8) | 80.2 (26.1) | 77.9 (24.5) | 78.3 (24.0)^*^ | 74.6 (42.3) |
| Saturated fat (g/day) | 26.4 (10.7) | 24.4 (9.67) | 23.1 (8.58) | 22.9 (8.41)^*^ | 23.3 (14.3) |
| Monounsaturated fat (g/day) | 35.6 (14.5) | 35.2 (12.3) | 34.2 (11.8) | 34.9 (11.6) | 32.2 (18.7) |
| Polyunsaturated fat (g/day) | 13.6 (6.03) | 13.4 (5.93) | 13.4 (5.77) | 13.3 (5.21) | 12.4 (7.92) |
| Omega-3 fatty acids (g/day) | 1.28 (0.77) | 1.53 (0.91) | 1.66 (1.01) | 1.84 (0.93)^*^ | 0.32 (0.76) |
| Total carbohydrate (g/day) | 213 (56.5) | 206 (54.3) | 207 (54.2) | 199 (48.4)^*^ | 209 (90.0) |
| Sugars (g/day) | 89.8 (32.6) | 89.6 (29.1) | 90.1 (28.9) | 89.7 (26.3) | 87.3 (35.5) |
| Dietary fiber (g/day) | 22.0 (7.04) | 23.5 (7.40) | 24.0 (7.03) | 24.7 (7.27)^*^ | 22.0 (10.4) |
| Vitamins |  |  |  |  |  |
| Carotenoids (mg/day) | 2.50 (1.59) | 3.03 (2.05) | 3.24 (2.03) | 3.77 (2.24)^*^ | 2.86 (1.80) |
| Thiamine (B_1_) (mg/day) | 1.24 (0.44) | 1.30 (0.44) | 1.36 (0.40) | 1.42 (0.40)^*^ | 1.23 (0.58) |
| Pyridoxin (B_6_) (mg/day) | 1.75 (0.49) | 1.92 (0.53) | 2.03 (0.52) | 2.15 (0.53)^*^ | 1.87 (0.85) |
| Vitamin C (mg/day) | 114 (62.1) | 132 (61.6) | 140 (61.6) | 151 (64.2)^*^ | 123 (73.7) |
| Vitamin E (µg/day) | 9.14 (4.35) | 9.67 (3.94) | 9.85 (4.31) | 10.1 (3.60)^*^ | 8.35 (4.55) |
| Minerals |  |  |  |  |  |
| Sodium (g/day) | 2.76 (1.15) | 2.67 (1.03) | 2.69 (0.95) | 2.66 (0.86) | 2.61 (1.54) |
| Potassium (g/day) | 2.95 (0.73) | 3.19 (0.74) | 3.36 (0.76) | 3.53 (0.76)^*^ | 3.12 (1.18) |
| Calcium (mg/day) | 854 (322) | 871 (286) | 886 (286) | 936 (264)^*^ | 869 (414) |
| Iron (mg/day) | 12.1 (3.48) | 12.6 (3.52) | 12.8 (3.24) | 13.1 (3.36)^*^ | 12.0 (5.86) |
| Selenium (µg/day) | 129 (51.1) | 134 (47.8) | 141 (46.8) | 143 (43.8)^*^ | 132 (67.2) |
| Values are means (standard deviations). *P value< 0.05 for differences in means (ANOVA) or proportions (Pearson’s chi-squared) across categories of the SEAD score | | | | | |
| ^a^ Sex-specific medians were used as the threshold for all food components, except wine. Quartile values of SEAD score: quartile 1, ≤2; quartile 2, 3; quartile 3, 4; quartile 4, ≥5. | | | | | |
| ^b^ The number of deaths/1000 person-years [95% confidence interval] for increasing quartiles of SEAD score were 26.8 [22.6, 32.1], 20.5 [17.2, 24.7], 17.9 [15.0, 21.6], and 16.9 [14.2, 20.2], respectively. | | | | | |

**Table S3**. Association between adherence to the Mediterranean Diet Adherence Screener (MEDAS) and the Alternate Healthy Eating Index (AHEI), and risk of all-cause death (n= 3165).

|  | **Hazard ratio [95% CI]** | | | |
| --- | --- | --- | --- | --- |
|  | **Deaths** | **Person-years** | **Model 1** | **Model 2** |
| **MEDAS score ^a^** |  |  |  |  |
| Per 1-SD increment | 646 | 32158 | 0.82 [0.75,0.91]*** | 0.89 [0.80,0.98]* |
| Quartiles |  |  |  |  |
| 1 (lower adherence) | 264 | 10347 | 1 | 1 |
| 2 | 172 | 7825 | 0.78 [0.63,0.96]* | 0.87 [0.70,1.09] |
| 3 | 112 | 6977 | 0.65 [0.51,0.84]*** | 0.73 [0.57,0.94]* |
| 4 (higher adherence) | 99 | 7009 | 0.62 [0.47,0.81]*** | 0.72 [0.55,0.95]* |
| **AHEI score ^b^** |  |  |  |  |
| Per 1-SD increment | 646 | 32158 | 0.80 [0.73,0.88]*** | 0.83 [0.76,0.92]*** |
| Quartiles |  |  |  |  |
| 1 (lower adherence) | 220 | 8033 | 1 | 1 |
| 2 | 151 | 7839 | 0.72 [0.57,0.91]** | 0.75 [0.59,0.95]* |
| 3 | 131 | 8417 | 0.58 [0.46,0.74]*** | 0.61 [0.48,0.78]*** |
| 4 (higher adherence) | 144 | 7869 | 0.57 [0.44,0.73]*** | 0.66 [0.51,0.85]** |
| * p<0.05; ** p<0.01; *** p<0.001; CI = confidence interval | | | | |
| ^a^ Quartile values of MEDAS score: quartile 1, ≤6; quartile 2, 7; quartile 3, 8; quartile 4, ≥9. Range: [2,13] | | | | |
| ^b^ Quartile values of AHEI score: quartile 1, ≤55.3; quartile 2, 55.3 to 61.9; quartile 3, 61.9 to 68.1; quartile 4, >68.1. Range: [20.7,96.6] | | | | |
| Model 1: Cox proportional hazards model adjusted for sex, age (years), educational level (primary or less, secondary, or university), and energy intake (kcal/day). | | | | |
| Model 2: As Model 1 and further adjusted for smoking status (never, former, or current), diabetes, cardiovascular disease, respiratory disease, musculoskeletal disease, cancer, and depression at baseline; and 3-year cumulative leisure-time physical activity (MET-hours/week), sedentary behavior (TV hours/day), and body mass index (kg/m^2^). | | | | |

**Table S4**. Association between adherence to the items of the Mediterranean Diet Adherence Screener (MEDAS) and the Alternate Healthy Eating Index (AHEI), and risk of all-cause death (n= 3165).

| **MEDAS score** | | | |
| --- | --- | --- | --- |
| **Items** | **Deaths** | **Person-years** | **Hazard ratio [95% CI] ^a^** |
| **Olive oil as the main fat for cooking** |  |  |  |
| No | 50 | 1413 | Ref. |
| Yes | 597 | 30745 | 0.67 [0.46,0.97]* |
| **Olive oil consumption** |  |  |  |
| <4 tablespoons/day | 604 | 29591 | Ref. |
| ≥4 tablespoons/day | 42 | 2567 | 0.97 [0.66,1.42] |
| **Vegetables** |  |  |  |
| <2 servings/day | 573 | 27370 | Ref. |
| ≥2 servings/day | 73 | 4788 | 0.89 [0.67,1.18] |
| **Fruit** |  |  |  |
| <3 pieces/day | 513 | 24249 | Ref. |
| ≥3 pieces/day | 133 | 7909 | 0.98 [0.80,1.21] |
| **Red meat or sausages** |  |  |  |
| ≥1 servings/day | 64 | 3168 | Ref. |
| <1 serving/day | 583 | 28990 | 1.03 [0.74,1.45] |
| **Animal fat** |  |  |  |
| ≥1 servings/day | 59 | 2495 | Ref. |
| <1 serving/day | 588 | 29663 | 0.77 [0.54,1.10] |
| **Sugar-sweetened beverages** |  |  |  |
| ≥1 cups/day | 70 | 2558 | Ref. |
| <1 cup/day | 576 | 29600 | 0.71 [0.53,0.95]* |
| **Wine** |  |  |  |
| <7 servings/week | 475 | 24116 | Ref. |
| ≥7 servings/week | 172 | 8042 | 0.95 [0.75,1.20] |
| **Legumes** |  |  |  |
| <3 servings/week | 537 | 27712 | Ref. |
| ≥3 servings/week | 109 | 4446 | 1.12 [0.85,1.47] |
| **Fish** |  |  |  |
| <3 servings/week | 255 | 10599 | Ref. |
| ≥3 servings/week | 391 | 21559 | 0.91 [0.75,1.10] |
| **Commercial pastries** |  |  |  |
| ≥2 servings/week | 328 | 14994 | Ref. |
| <2 servings/week | 318 | 17164 | 0.93 [0.78,1.11] |
| **Nuts** |  |  |  |
| <3 servings/week | 516 | 22176 | Ref. |
| ≥3 servings/week | 130 | 9981 | 0.71 [0.58,0.89]** |
| **Preferring white meat over red meat** |  |  |  |
| No | 462 | 24365 | Ref. |
| Yes | 185 | 7793 | 1.22 [0.99,1.51] |
| **Traditional sauce of tomatoes, garlic, onion, or leeks sautéed in olive oil** |  |  |  |
| <2 servings/week | 64 | 1674 | Ref. |
| ≥2 servings/week | 582 | 30484 | 0.61 [0.44,0.83]** |

**Table S4 (cont.)**

| **AHEI score** | | | |
| --- | --- | --- | --- |
| **Items** | **Deaths** | **Person-years** | **Hazard ratio [95% CI] ^a^** |
| **Vegetables** |  |  |  |
| Per 1-point increment of the item | 646 | 32158 | 0.96 [0.91,1.02] |
| **Fruit** |  |  |  |
| Per 1-point increment of the item | 646 | 32158 | 0.97 [0.94,1.01] |
| **Whole grains** |  |  |  |
| Per 1-point increment of the item | 646 | 32158 | 0.94 [0.83,1.07] |
| **Sugar-sweetened beverages and fruit juice (reverse scoring) ^b^** |  |  |  |
| Per 1-point increment of the item | 646 | 32158 | 0.97 [0.94,0.99]* |
| **Nuts and legumes** |  |  |  |
| Per 1-point increment of the item | 646 | 32158 | 0.97 [0.94,1.01] |
| **Red and processed meat (reverse scoring) ^b^** |  |  |  |
| Per 1-point increment of the item | 646 | 32158 | 1.02 [0.99,1.06] |
| **Trans fat (reverse scoring) ^b^** |  |  |  |
| Per 1-point increment of the item | 646 | 32158 | 0.95 [0.88,1.03] |
| **Long-chain (n-3) fats (EPA + DHA)** |  |  |  |
| Per 1-point increment of the item | 646 | 32158 | 0.96 [0.92,1.00]* |
| **PUFA** |  |  |  |
| Per 1-point increment of the item | 646 | 32158 | 0.98 [0.94,1.04] |
| **Sodium (reverse scoring)^b^** |  |  |  |
| Per 1-point increment of the item | 646 | 32158 | 0.97 [0.93,1.01] |
| **Alcohol (moderate)** |  |  |  |
| Per 1-point increment of the item | 646 | 32158 | 0.96 [0.94,0.99]** |
| CI = confidence interval, EPA = Eicosapentanoic acid, DHA = Docosahexanoic acid, PUFA = Poliunsaturated fatty acids. | | | |
| * p<0.05; ** p<0.01; *** p<0.001 | | | |
| ^a^ Cox proportional hazards model as Model 2 in Supplemental Table 3, adjusted for sex, educational level (primary or less, secondary, or university), age (years), smoking status (never, former, or current), diabetes, cardiovascular disease, respiratory disease, musculoskeletal disease, cancer, and depression at baseline; and 3-year cumulative leisure-time physical activity (MET-hours/week), sedentary behavior (TV hours/day), body mass index (kg/m^2^), and energy intake (kcal/day). | | | |
| ^b^ Higher scores indicate a lower intake of the item. | | | |

**Figure S1.** Participants’ flow chart.

Participants ≥60 years old in the ENRICA study (2008-2010)

n = 3483

EXCLUDED FROM ANALYSES (total = 318):

Inadequate data on diet (total = 252)

- No diet history (n = 239)
- Implausible energy intake (n = 13)
  - ≤800 or ≥5000 kcal/day in men
  - ≤500 or ≥4000 kcal/day in women

Missing data on potential confounders (total = 254)

- Educational level (n = 9)
- Tobacco smoking (n = 11)
- Recreational physical activity (n = 9)
- Sedentary behavior (n = 43)
- Body mass index (n = 209)

January 31^th^, 2020

**Analytical sample**

**n = 3165**

Note that one individual may lack data in more than one variable.

**Figure S2.** Association between adherence to the Mediterranean Diet Adherence Screener (MEDAS) and the Alternate Healthy Eating Index (AHEI), and risk of all-cause death.

Plotted values are Hazard Ratios (95% confidence intervals) from a Cox proportional hazards model as Model 2 in Supplemental Table 3, adjusted for sex, educational level (primary or less, secondary, or university), age (years), smoking status (never, former, or current), diabetes, cardiovascular disease, respiratory disease, musculoskeletal disease, cancer, and depression at baseline; and 3-year cumulative leisure-time physical activity (MET-hours/week), sedentary behavior (TV hours/day), body mass index (kg/m^2^), and energy intake (kcal/day).

The restricted cubic spline knots are located at 6.5, 7.5, and 8.5 points of adherence to the MEDAS and at 55.3, 61.9, and 68.1 points of adherence to the AHEI.
